# A review of ammonia-oxidizing bacteria and archaea in Chinese soils

**DOI:** 10.3389/fmicb.2012.00296

**Published:** 2012-08-21

**Authors:** Ju-Pei Shen, Li-Mei Zhang, Hong J. Di, Ji-Zheng He

**Affiliations:** ^1^State Key Laboratory of Urban and Regional Ecology, Research Center for Eco-Environmental Sciences, Chinese Academy of SciencesBeijing, China; ^2^Centre for Soil and Environmental Research, Lincoln UniversityLincoln, New Zealand

**Keywords:** ammonia-oxidizing archaea, ammonia-oxidizing bacteria, Chinese soil, distribution, fertilization, nitrification, inhibition

## Abstract

Ammonia (NH_3_) oxidation, the first and rate-limiting step of nitrification, is a key step in the global Nitrogen (N) cycle. Major advances have been made in recent years in our knowledge and understanding of the microbial communities involved in ammonia oxidation in a wide range of habitats, including Chinese agricultural soils. In this mini-review, we focus our attention on the distribution and community diversity of ammonia-oxidizing bacteria (AOB) and ammonia oxidizing archaea (AOA) in Chinese soils with variable soil properties and soil management practices. The niche differentiation of AOB and AOA in contrasting soils have been functionally demonstrated using DNA-SIP (stable isotope probing) methods, which have shown that AOA dominate nitrification processes in acidic soils, while AOB dominated in neutral, alkaline and N-rich soils. Finally, we discuss the composition and activity of ammonia oxidizers in paddy soils, as well as the mitigation of the greenhouse gas nitrous oxide (N_2_O) emissions and nitrate leaching via inhibition of nitrification by both AOB and AOA.

## Introduction

Nitrification is a major process in the nitrogen (N) cycling, including a two-step process, the oxidation of ammonia to nitrite and subsequently nitrite to nitrate (Prosser, 1989). Ammonia oxidation, the first and rate-limiting step of nitrification, has been studied widely because of its ecological significance in the global N cycle and environmental implications (Kowalchuk and Stephen, 2001). For over a century, ammonia-oxidizing bacteria (AOB) were considered to be the main driver for ammonia oxidation. This view was recently challenged, however, by the discovery of ammonia-oxidizing archaea (AOA) (Venter et al., 2004; Könneke et al., 2005; Treusch et al., 2005). The first study showing the potential importance of AOA in soils was carried out in different European soils, and revealed that AOA were clearly dominant among ammonia oxidizers (Leininger et al., 2006). Subsequent studies have confirmed the widespread distribution of AOA and their numerical dominance over AOB in a number of aquatic and terrestrial environments (e.g., He et al., 2007; Dang et al., 2008). The importance of AOA in ammonia oxidation has attracted intense research attention in recent years (Nicol and Schleper, 2006; Prosser and Nicol, 2008). Soil molecular biology techniques such as DNA-based stable isotope probing (DNA-SIP) and transcription analysis have been utilized in order to elucidate the contribution of AOA to ammonia oxidation and thereby understand the link between AOA abundance, diversity and ecosystem function (Tourna et al., 2008; Jia and Conrad, 2009; Offre et al., 2009). Erguder et al. (2009) reviewed environmental factors that might affect the distribution of AOA and suggested that environmental factors might shape specific niches of AOA and their contribution to nitrification, particularly in low-nutrient and low pH environments. More recently, the successful cultivation of three AOA isolates from soil environments (Jung et al., 2011; Lehtovirta-Morley et al., 2011; Tourna et al., 2011) and evidence from SIP experiments have confirmed the activity and function of AOA in nitrification in low-N input and low pH soils (Zhang et al., 2010, 2012). However, the relative importance of AOB and AOA in ammonia oxidation is still not fully understood, and their relative contribution to nitrification may vary, depending on soil conditions.

China represents an enormous agricultural landscape with diverse soil types and pH. Research on nitrification has received much attention since the 1980's due to large increases in the use of ammonia-based N fertilizers and their associated environmental consequences. The discovery of archaeal ammonia oxidation also stimulated major research interest on ammonia oxidizers in Chinese soils. In this mini-review, we will summarize recent research findings on community composition and distribution of ammonia oxidizers in Chinese soils, environmental factors that affect the abundance and activity of AOB and AOA, and discuss the relative importance of AOB and AOA in ammonia oxidation in different soil systems.

## Abundance and composition of AOB and AOA in chinese soils

Biological ammonia oxidation occurs when the multimeric enzyme ammonia monooxygenase (AMO) converts ammonia to hydroxylamine. The alpha (A) subunit of the AMO enzyme is encoded by the *amo*A gene, variants of which are commonly found in both bacteria and archaea. With the availability of culture-independent molecular ecology techniques such as denaturing gradient gel electrophoresis (DGGE), terminal restriction fragment length polymorphism (T-RFLP), cloning, sequencing, and quantitative PCR in China, new insights into the diversity and distribution of AOB and AOA targeting the *amo*A genes in various Chinese soils have been rapidly achieved during the last five years. The abundance and composition of AOB and AOA in acidic red soils (pH 3.7–6.0), for example, were first investigated at a long-term field experiment station located at Qiyang (Hunan province, southern China), which had received continuous fertilization practice for 16 years (He et al., 2007). In soil samples taken from all eight fertilization treatments, copy numbers of AOA *amo*A gene variance were greater than those of AOB *amo*A genes, with the ratios of AOA to AOB ranging from 1–12 (He et al., 2007). These results supported the observation by Leininger and colleagues (2006) that AOA outnumbered AOB in agricultural soils. A similar trend was observed in different study of alkaline agricultural soil with pH ranging from 8.3 to 8.7 (Shen et al., 2008). These studies of Chinese soils (He et al., 2007; Shen et al., 2008) were the first to demonstrate the presence of both AOB and AOA in pH-contrasting agricultural soils. Subsequently, comparative analyses of AOB and AOA in China covered a large geographic range of agricultural soils with different management regimes (Chen et al., 2008; Wang et al., 2009; Ying et al., 2010; Yao et al., 2011; Zhang et al., 2012), as well as various habitats, including grassland (Shen et al., 2011) and paddy (Chen et al., 2010) soils. A general trend identifiable from these studies is that archaeal *amo*A genes are more abundant than bacterial *amo*A genes in most of the soils studied, with the exception of two long-term fertilization treatments, in which the ratios of AOA to AOB *amo*A genes were 0.25 and 0.39 (Wu et al., 2011).

**Figure 1:**
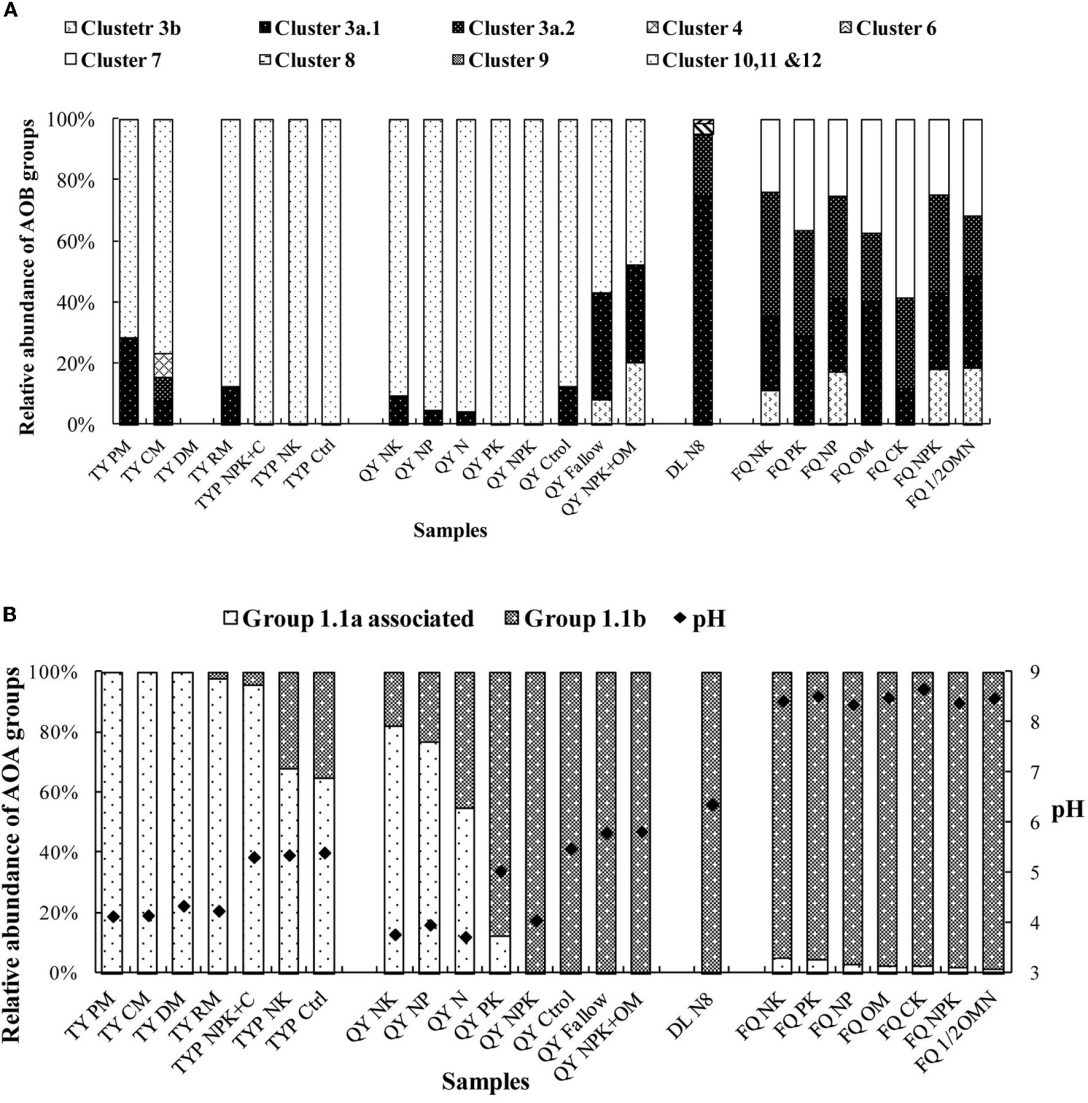
**Relative abundances of bacterial and archaeal ammonia-oxidizer groups in selected Chinese soils. (A)** AOB community composition; **(B)** AOA community composition. Sample names are as designated in the “Sample Name” column of Table 1.

In order to obtain a comprehensive picture of the distribution of AOB and AOA in different Chinese soils, we reanalyzed our partially published data on the community composition of AOA and AOB in 23 soil samples from five sites (Table 1). The AOB community was mainly dominated by *Nitrosospira amo*A cluster 10, 11 and 12 in acidic red soils, while *Nitrosospira amo*A clusters 3a.1, 3a.2 and *Nitrosomonas amo*A cluster 6 and 7 are mainly distributed in alkaline and neutral soils (Figure 1A). Archaeal *amo*A sequences retrieved from these soils fell into two major lineages, group 1.1a-associated and group 1.1b (Figure 1B). Correlation analysis showed that the relative abundance of group 1.1a was negatively correlated with soil pH (*r* = −0.553, *n* = 23, *p* < 0.05) whereas that of group 1.1b was positively correlated with pH (*r* = 0.357, *n* = 23, *p* < 0.05). Meta-analysis and high-throughput sequencing analysis by Gubry-Rangin et al. (2011) showed that soil pH was the only measured physicochemical property that significantly influenced the community structure of AOA. The sequences used in this study were obtained from soils with a wide range of pH (i.e., 3.7–8.8) similar to the pH range in the Chinese soils in our study. Subsequent studies of Chinese soils have made further contributions to our knowledge of the global distribution patterns of AOB and AOA and key driving factors.

**Table 1:** **Physical and chemical properties of the twenty-three soils used for the meta-analysis**.

***^a^*Location and experimental design**	**Soil type**	**Sample name**	**Treatment**	**pH**	**Organic matter (mg kg^−1^)**	**NH^+^_4_-N (mg kg^−1^)**	**NO^−^_3_-N (mg kg^−1^)**	**Longitude (°)**	**Latitude (°)**	**Altitude (m above sea level)**	**Mean temperature (°C)**	**Reference**
Taoyuan(TY),	Acidic	TY PM	Pine plantation	4.1	11.6	0.0	18.5	111.433	28.917	110	16.5	Ying et al., 2010
Hunan,	red soil	TY CM	Cropland	4.1	9.5	2.4	45.3	111.433	28.917	110	16.5	
Land utilization		TY DM	Degradation	4.3	14.0	1.6	0.5	111.433	28.917	110	16.5	
patterns		TY RM	Restoration	4.2	16.5	6.2	13.3	111.433	28.917	110	16.5	
Taoyuan(TYP), Hunan, Different fertilizations	Acidic paddy soil	TYP NPK+C	^b^N, P, K fertilizers and organic matter	5.3	41.6	85.9	0.9	111.433	28.917	110	16.5	Chen et al., 2011
		TYP NK	N plus K	5.3	30.4	55.8	0.7	111.433	28.917	110	16.5	
		TYP CK	No fertilization	5.4	29.2	27.1	0.6	111.433	28.917	110	16.5	
Qiyang(QY), Hunan, Different fertilizations	Acidic red soil	QY NK	N plus K fertilizers	3.8	14.3	15.5	35.2	111.867	26.750	100	18.0	He et al., 2007
		QY NP	N plus P fertilizers	4.0	16.4	13.4	21.5	111.867	26.750	100	18.0	
		QY N	N fertilizer	3.7	15.2	21.0	43.6	111.867	26.750	100	18.0	
		QY PK	P plus K fertilizers	5.0	14.6	19.7	6.0	111.867	26.750	100	18.0	
		QY NPK	N, P, K fertilizers	4.0	16.9	27.8	8.7	111.867	26.750	100	18.0	
		QY CK	No fertilization	5.5	13.6	19.1	5.1	111.867	26.750	100	18.0	
		QY Fallow	Fallow	5.8	13.7	13.4	0.7	111.867	26.75	100	18.0	
		QY NPK+OM	N, P, K fertilizers and organic matter	5.8	21.3	17.7	18.7	111.867	26.75	100	18.0	
Duolun(DL), Inner Mongolia	Chestnut soil	DL N8	Urea fertilizer	6.4	46.5	3.0	7.6	116.289	42.041	1324	2.1	Shen et al., 2011
Fengqiu(FQ), Henan, Different fertilizations	Sandy loam soil	FQ NK	N plus K fertilizers	8.4	7.4	5.4	35.2	114.400	35.000	67.5	13.9	Shen et al., 2008
		FQ PK	P plus K fertilizers	8.5	8.4	4.7	2.4	114.400	35.000	67.5	13.9	
		FQ NP	N plus P fertilizers	8.3	9.5	4.3	33.4	114.400	35.000	67.5	13.9	
		FQ OM	Organic matter	8.5	16.7	5.5	4.9	114.400	35.000	67.5	13.9	
		FQ CK	No fertilization	8.7	7.3	6.1	6.5	114.400	35.000	67.5	13.9	
		FQ NPK	N,P,K fertilizers	8.4	9.8	4.7	22.6	114.400	35.000	67.5	13.9	
		FQ 1/2OMN	1/2(N,P,K fertilizers plus organic matter)	8.5	12.8	5.2	10.9	114.400	35.000	67.5	13.9	

a Sample name was designated as the sampling location, followed by the treatment.

b Fertilization abbreviation: chemical fertilizer nitrogen (N), phosphorus (P) and potassium (K). “-” no data.

## Activity and function of AOB and AOA in the soils

In different soil systems, AOB and AOA communities respond differently to changes in environmental factors. For example, in acidic red soils from Qiyang station, although the potential nitrification rate (PNR) was positively correlated with both AOB and AOA abundance, a pronounced shift of community composition was only observed in AOA but not in AOB in response to the long-term fertilization treatments (He et al., 2007). In contrast, in alkaline soils from the Fengqiu experiment station (FQ, in northern China) which had a fertilization practices similar to the Qiyang station, it was observed that the AOB, and not AOA, community composition varied significantly between the different fertilization treatments (Shen et al., 2008). Additionally, PNR at this site was positively correlated with AOB, but not AOA *amo*A gene copy numbers (Shen et al., 2008). These studies suggest that AOA may be more active than AOB in acidic soils, whereas it may be the opposite in alkaline soils. Two recent studies, both examining of Chinese tea orchard soils, further demonstrated the dominant role of AOA in ammonia oxidation in acidic soils, either* in situ* in a field study (Yao et al., 2011) or in a microcosm experiment (Zhang et al., 2012). The study also demonstrated a significant relationship between nitrification potential and community abundance of AOA, but not AOB (Yao et al., 2011). Zhang et al. (2012) found significant assimilation of soil inorganic carbon by AOA, but not by AOB, concurrent with accumulation of soil nitrate, thus providing direct evidence of AOA contribution to autotrophic nitrification in acidic soil microcosms. These results and those from upland acidic soils would suggest that AOA communities have greater adaptability to low pH environments than their AOB counterparts. This speculation has been recently confirmed by cultivation and characterization of an obligate acidophilic thaumarchaeal ammonia oxidizer from a nitrifying acidic soil (Lehtovirta-Morley et al., 2011).

The study on alkaline soils from the Fengqiu experiment station found that differences in fertilization regimes did not alter the abundance of AOA, but rather resulted in an increase in AOB abundance in soils receiving N fertilizer (Shen et al., 2008). This finding is consistent with the observation that N fertilizer amendment changed the abundance and composition of AOB, but had no significant effect on AOA community composition or abundance, in a semi-arid temperate grassland soil with neutral pH (Shen et al., 2011). This suggests that N fertilization provides a growth advantage for AOB relative to AOA, and that AOB may actively be involved in the nitrification of alkaline and N-rich neutral soils. Xia et al. (2011) further demonstrated that AOB were the primary drivers of ammonia oxidation in an alkaline Chinese soil in a microcosm study which included weekly inorganic N additions. These results support earlier findings in pH-neutral N-rich New Zealand grassland soils (Di et al., 2009), in which AOB were demonstrated to be more important than AOA for ammonia oxidation. These studies together confirm that the roles of AOB and AOA in soil ammonia oxidation may vary dependent upon soil conditions, and can thus be hypothesized to occupy different ecological niches based on N availability and pH (He et al., 2012).

## AOB and AOA in chinese paddy soils

China is one of the world's largest rice producers, accounting for about one quarter of the world's rice paddy cultivation area. The paddy habitat is a representative wetland system for analysis of biogeochemistry and microbiological processes due to frequent oxic/anoxic alternations. Oxygen is transported from the atmosphere into the roots through the rice porous tissue, results in an oxic environment capable of supporting nitrification within the rice rhizosphere (Arth et al., 1998). Although nitrification in paddy soil has been studied widely, the underlying microbial contributions to nitrification process are poorly understood.

Microcosm experiments by Chen et al. (2008) first shed light on the presence of abundant AOA in Chinese paddy soils. This study demonstrated that the abundance of AOA was greater than AOB both in bulk and rhizosphere soils with or without rice plants with the AOA to AOB ratios ranging from 1.2 to 69.3. The numerical predominance of AOA over AOB in paddy soils was further confirmed by a study on acidic field paddy soils with different long-term fertilization treatments (Chen et al., 2011) or on different paddy soil types (Chen et al., 2010). Rice cultivation strongly stimulated AOA abundance, but not AOB abundance, as determined by quantification of *amoA* gene copies (Chen et al., 2008). Empirical evidence suggests that AOA are able to withstand a wide range of oxygen levels (Erguder et al., 2009), and bioinformatic analysis of AOA genomes indicates the potential for mixotrophic metabolism, i.e., alternation between chemoautotrophy (nitrification) and heterotrophic growth of rice root exudates (Walker et al., 2010; Pester et al., 2011).

The most commonly used N fertilizer on paddy soils in China is urea. Treatment of rice paddy soil from Northern China (pH 6.9) with different amounts of urea-N had a clear effect on the structure of AOB community, while the AOA structure remained stable during the period of investigation (Wang et al., 2009). Similarity, a significant shift in community structure and an increase in abundance were observed for AOB, but not AOA, in a paddy soil (pH 6.4) from a long-term fertilization field experiment in Changshu, Jiangsu Province of central China (Wu et al., 2011). Ke and Lu (2012) also observed that the composition of the AOA community was not responsive to changes in the soil environment and N amendment in a microcosm study of acidic (pH 5.7) and alkaline (pH 8.3) rice field soils. In contrast, one study demonstrated the apparent stimulation of AOA growth in rice paddy soil at low pH (Chen et al., 2011). Given the small number of studies investigating nitrifier communities in rice paddy soil, it is still not possible to draw conclusions as to whether soil pH is a selective factor for the niche separation of AOA and AOB in this waterlogged and high N-input soil system. Further research on nitrification in rice paddy soils is clearly required.

## Inhibition of ammonia oxidation by AOB and AOA

From an agricultural perspective, nitrification represents both a potential source of N loss, by which N fertilizer becomes unavailable for crop plant nutrition, and a source of greenhouse gas emission through generation of nitrous oxide (N_2_O) gas. The development of nitrification inhibitors has therefore played an instrumental role in controlling ammonia oxidation rates, and thus reducing N losses through nitrate leaching and N_2_O emissions (Prosser, 1989; Di and Cameron, 2002). In this review, emphasis is placed on the effects of the nitrification inhibitors dicyandiamide (DCD) and acetylene (C_2_H_2_) on AOB and AOA community composition, abundance and function. DCD is a non-volatile commercially-available compound which inhibits nitrification by covalently binding to the active site of, and thereby inactivating the AMO enzyme (Amberger, 1989; McCarty and Bremner, 1989). Significant inhibition by DCD on AOA, but not on AOB, was recently observed in a microcosm study of acidic Chinese acidic soil (pH < 4.5) (Zhang et al., 2012). The same study also demonstrated that AOA were functionally dominant over AOB in the soil under investigation, confirming effective inhibition of AOA nitrification by DCD. In contrast, Di and co-workers (2009, 2010) observed that AOB were positively selected for by application of high rates of ammonium substrates, and that this growth was significantly inhibited by the application of DCD, in urine-rich grazed pasture soil. In one study, application of DCD decreased NO^−^_3_ leaching by 59% and N_2_O emissions by 64% from animal urine patches in N-rich New Zealand grassland soils (Di et al., 2010). The variable susceptibility of AOB and AOA communities to inhibition by DCD is noteworthy. The predicted protein structure of the AMO enzyme from the marine archaeon Candidatus *Nitrosopumilus maritimus* suggested that ammonia oxidation to nitrite occurs via a nitroxyl intermediate, rather than via the hydroxylamine intermediate utilized by bacterial AMO (Walker et al., 2010). A natural conclusion from this evidence is that archaeal and bacterial AMO enzymes may by merit of their structural and/or functional differences respond differently to DCD inhibition, although further investigation is clearly required before conclusions may be drawn. In general, DCD is considered an effective inhibitor of nitrification by merit of its ability to inactivate the AMO enzyme produced by the actively nitrifying microbial community, regardless of whether the activity is dominated by AOA or AOB. An improved understanding here will help to enhance our ability to regulate the soil nitrification process and then mitigate N_2_O emissions.

Another important nitrification inhibitor is acetylene (C_2_H_2_), which has been widely used as a suicide inhibitor of AMO (De Boer et al., 1991; Offre et al., 2009). Coupling molecular analysis with C_2_H_2_ inhibition of nitrification in an alkaline Chinese soil, Xia et al. (2011) revealed that the growth of AOA and AOB were both inhibited by C_2_H_2_, although the inhibition of AOB growth was greater. Interestingly, various studies using C_2_H_2_ as an inhibitor of nitrification have yielded different results with reference to the microbial groups that dominate ammonia oxidation. For example, ammonia oxidation in microcosms containing a Scottish agricultural soil was dominated by AOA whose growth was suppressible by C_2_H_2_-treatment (Offre et al., 2009; Zhang et al., 2010). In contrast, Jia and Conrad (2009) showed through C_2_H_2_ treatment of German agricultural soils that AOB ammonia oxidation were more functionally important than AOA in the same soils.

Nitrifier denitrification is considered a significant source of soil N_2_O emissions under aerobic conditions (Wrage et al., 2005; Kool et al., 2011). Although long thought to be mediated primarily by AOB, it is unclear whether AOA also contribute to soil N_2_O emissions. A recent report analyzing stable isotopic signatures of N_2_O emissions showed that AOA may be largely responsible for N_2_O emissions from marine environments (Santoro et al., 2011), yet more research is required in order to increase understanding of this process in soil habitat.

## Conclusions and perspectives

Studies on the ecological distribution and community dynamics of AOB and AOA across a wide range of soil habitats and management regimes in China have demonstrated broad physiological diversity and distinct ecophysiology under contrasting soil and climatic conditions. However, it remains to be ascertained whether different functions, or possible functional redundancy, of phylogenetically distinct AOB and archaeal groups exist under different environmental conditions. In order to better understand soil N dynamics, research on the spatial and temporal variations of AOB and AOA activity and their unique contributions to nitrification is urgently needed, particularly in water-logged rice paddy soils where biogeochemical activity is high due to frequent oxic/anoxic shifts. Increased magnitude and frequency of N fertilizer application to agricultural soils stimulates the emissions of N_2_O, a potent greenhouse gas. Integrated investigations of AOA and AOB communities using complementary methodologies are expected to facilitate identification of nitrification and denitrification activities of archaeal and bacterial ammonia oxidizers under different soil conditions. In order to reduce greenhouse gas emissions and nutrient loss from agricultural soils, it is imperative to gain a better understanding of the microbial communities involved and their specific contributions to nitrification and soil N cycling in general. This will be critical for increasing our ability to develop better strategies for N cycle management and to improve the N use efficiency, while at the same time minimizing adverse environmental impacts.

### Conflict of interest statement

The authors declare that the research was conducted in the absence of any commercial or financial relationships that could be construed as a potential conflict of interest.
